# Analytical approximation for invasion and endemic thresholds, and the optimal control of epidemics in spatially explicit individual-based models

**DOI:** 10.1098/rsif.2020.0966

**Published:** 2021-03-31

**Authors:** Yevhen F. Suprunenko, Stephen J. Cornell, Christopher A. Gilligan

**Affiliations:** ^1^ Department of Plant Sciences, University of Cambridge, Downing Street, Cambridge CB2 3EA, UK; ^2^ Institute of Infection, Veterinary, and Ecological Sciences, University of Liverpool, Liverpool L69 7ZB, UK

**Keywords:** basic reproduction number, endemic equilibrium, spatially explicit systems, epidemic control, optimal control, lockdown exit strategy

## Abstract

Computer simulations of individual-based models are frequently used to compare strategies for the control of epidemics spreading through spatially distributed populations. However, computer simulations can be slow to implement for newly emerging epidemics, delaying rapid exploration of different intervention scenarios, and do not immediately give general insights, for example, to identify the control strategy with a minimal socio-economic cost. Here, we resolve this problem by applying an analytical approximation to a general epidemiological, stochastic, spatially explicit SIR(S) model where the infection is dispersed according to a finite-ranged dispersal kernel. We derive analytical conditions for a pathogen to invade a spatially explicit host population and to become endemic. To derive general insights about the likely impact of optimal control strategies on invasion and persistence: first, we distinguish between ‘spatial' and ‘non-spatial' control measures, based on their impact on the dispersal kernel; second, we quantify the relative impact of control interventions on the epidemic; third, we consider the relative socio-economic cost of control interventions. Overall, our study shows a trade-off between the two types of control interventions and a vaccination strategy. We identify the optimal strategy to control invading and endemic diseases with minimal socio-economic cost across all possible parameter combinations. We also demonstrate the necessary characteristics of exit strategies from control interventions. The modelling framework presented here can be applied to a wide class of diseases in populations of humans, animals and plants.

## 1. Introduction

For emerging diseases, such as COVID-19 [1–7], and re-occurring diseases typified by annual influenza [8–13], there is usually a large variety of control measures of different effectiveness and different socio-economic cost. Given limited resources, it is crucially important to have a good understanding of what combination of control measures would constitute an optimal control strategy [14–25], and to be able to identify such an optimal strategy quickly. This requires two key elements: (i) an accurate description of epidemics that allows a general understanding and rapid exploration of different scenarios and (ii) a reliable model of control measures to quantify their relative cost and impact on epidemics.

Rapid and reliable identification of an optimal control strategy for an emerging epidemic is still a major challenge mainly due to theoretical and computational difficulties in describing the spatial stochastic spread and persistence of diseases. Simulations of individual-based models [15,25–28] and network models [3,29–31] often are highly detailed and may provide reliable predictions, but can take a long time to provide results. Complex simulation models do not allow quick exploration of different epidemic and control scenarios. Analytical models, in contrast, can provide insights about epidemic principles, e.g. regarding the invasion threshold for a pathogen [32–39], or the level of vaccination needed to stop the spread of the pathogen in a population [40,41]. However, most analytical insights are based on classical, non-spatial epidemiological models [32] that do not account for spatial dynamics. Analytical approximations in the form of ‘moment closure' [42–44] for individual-based models and ‘pair-approximation' [29] in lattice and networks models currently have two important limitations. One limitation is that the approximation schemes are uncontrolled and not guaranteed to provide an exact result in any particular limit [45,46]. An alternative more reliable approximation scheme introduced in Ovaskainen & Cornell [47] and Ovaskainen *et al.* [48] for spatial point processes and individual-based models provides asymptotically exact results when interactions between individuals are sufficiently long-ranged. The approximation can be applied to a wide class of individual-based models [49], but until now has rarely been applied to epidemiological problems (but see [50] for applications to spatially explicit metapopulations).

A second limitation of moment closure and pair approximation techniques is revealed when estimating the invasion threshold for infectious diseases [43,44]. Bolker, in particular, has shown [43] that spatial moment equations cannot be used to compute invasion eigenvalues for a dynamic system such as an epidemic. Instead, as stated in Brown & Bolker [44], ‘to compute the threshold, one needs to compute the spatial structure of the initial phase of the (potential) epidemic'. Accordingly, it is necessary to estimate or assume the local spatial structure [29,44,50] and use that to determine whether a global invasion can proceed. The invasion threshold is therefore estimated not at the start of the epidemic, but at a certain later time when the so-called ‘pseudoequilibrium' at the local scale is achieved [44]. Examples of such estimates can be found in individual-based models [44], in network-based models [29] as well as in spatially explicit metapopulation models [50].

In this paper, we adapt the method of Cornell *et al*. [49] to present a reliable analytical description of epidemics in spatially explicit epidemiological models. Our solution permits rapid exploration of the relative impact of different control scenarios with a view to the identification of the optimal control strategy. Our approach also overcomes the limitations associated with uncontrolled approximation schemes. By exploiting the fact that the invasion is a localized phenomenon when interactions are localized, we estimate the invasion threshold at the start of an epidemic. In the case when re-infection is possible, we also quantify the fraction of infected individuals at the endemic equilibrium. We show that our analytical model allows a classification of a wide class of control measures into spatial and non-spatial measures based on their influence on spatially explicit transmission of infection. This classification leads to a quantified estimate of the impact of control measures on disease incidence, and, therefore, to the identification of the optimal control strategy subject to the known relative cost of different control measures. This paper was motivated by the current COVID-19 pandemic [51,52], and, therefore, for numerical results, we used parameters of the COVID-19 epidemic in the UK as the most relevant example. However, our approach is applicable to a wide class of diseases in populations of humans, animals and plants.

## 2. Methods

### 2.1. Model

We use the modelling framework introduced in Cornell *et al*. [49]. The modelling framework [49] formulates individual-based models by stochastic spatio-temporal point processes, derives an exact expression for the moment equations to all orders and, using a perturbation scheme [47,48], provides equations that reliably approximate the effects of space and stochasticity.

We consider the simplest example of a spatially explicit host population where all individuals are stationary and distributed randomly (Poisson process). Within this modelling framework, individuals are distributed with a constant spatial density, *n*, in infinite two-dimensional Euclidean space. However, one can think of this as representing a population of total size *N* distributed over a large area *A*, so that *n* = *N*/*A*. Individuals can be susceptible, infected or recovered, described by their expected densities *Q_S_*, *Q_I_* and *Q_R_*, respectively. Correspondingly, for a real-life population of total size *N*, these densities are calculated as *Q_S_* = *S*/*A*, *Q_I_* = *I*/*A* and *Q_R_* = *R*/*A*, where *S*, *I* and *R* denote the total number of individuals in the susceptible, infected or recovered compartments.

**Figure 1.**
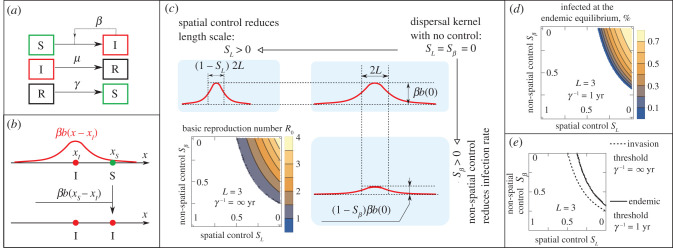
Modelling the impact of spatial and non-spatial control measures on disease incidence in spatially explicit individual-based models. (*a*) Epidemiological compartments and transitions in the SIR(S) model. (*b*) Spatially explicit transmission of a pathogen in the model is defined by the normalized dispersal kernel *b*(*x*) shown here as a red curve centred around the location *x_I_* of an infected individual. A susceptible individual located at *x_S_* becomes infected with the rate *βb*(*x_I_* − *x_S_*) where *x_I_* − *x_S_* is the distance between individuals; the process of infection is illustrated by a vertical line, the resulting state is shown in the bottom part of the panel. (*c*) Spatial and non-spatial control measures are defined by their impact on the dispersal kernel *b*(*x*); their impact on the basic reproduction number *R*_0_ in an SIR model. (*d*) The impact of control measures on the percentage of infected individuals $100\text{\%}\times Q_{I}^{\mathrm{endemic}}/n$ at the endemic equilibrium in an SIRS model. (*e*) Invasion threshold *R*_0_ = 1, and endemic threshold $Q_{I}^{\mathrm{endemic}}=0$ are not identical in spatially explicit systems with finite range, *L*, of dispersal kernels. In both SIR and SIRS models, individuals are stationary and distributed randomly with an average density, *n*, of one individual per unit area, *n* = 1. *L* is measured in units where the population density is equal to 1.

Individuals change type by one of the following transitions (figure 1*a*): one infected individual interacting with *N* susceptible individuals creates *βN* newly infected individuals per unit of time, where *β* is the infection rate per contact; each infected individual becomes a recovered and immune individual with rate *μ*; the immunity to a pathogen disappears with rate *γ*, i.e. the average duration between the end of infectiousness and the loss of immunity is 1/*γ*. The SIRS model reduces to a SIR model, when *γ* = 0. For diseases with confirmed short-lasting immunity, one should use *γ* > 0, and the model corresponds to an SIRS epidemiological model.

The infection transmission in the model is spatially explicit: the infection rate *β* is distributed in space according to a dispersal kernel, *b*(*r*). This means that an infection of a susceptible individual located at the point *x_S_* by an infected individual located at the point *x_I_* will occur with the rate *βb*(*r*), where *r* is the distance between individuals, *r* = |*x_S_* − *x_I_*| (figure 1*b*). The dispersal kernel *b*(*r*) is defined as a normalized ($\int_{0}^{\infty }2\pi rb(r)\;dr=1$), non-increasing, non-negative function of the distance *r* between infected and susceptible individuals. Moreover, the kernel, *b*(*r*), is a finite-ranged kernel characterized by the length scale *L* (e.g. a radius of the Tophat kernel, or a standard deviation of the Gaussian kernel). Thus, within the spatially explicit model with finite-ranged kernel *b*(*r*), one infected individual has direct contact only with a finite number of susceptible individuals and does not contact individuals located at distances much larger than *L*. By contrast, in non-spatial models because of homogeneous mixing each infected individual can infect each susceptible individual in a population directly with the same constant infection rate. In our model, which considers individuals in infinite space, homogeneous mixing is obtained in the limit *L* → ∞. In such a case, when *L* = ∞, our model provides the same results as a corresponding non-spatial epidemiological model; see electronic supplementary material, Supplementary Note S1.

Dynamical equations for the expected densities *Q_S_*, *Q_I_* and *Q_R_* are constructed from equations presented in Cornell *et al.* [49] for the spatial point process ‘Infection’ (to model the dispersal of infection, i.e. when infected individuals infect susceptible individuals) and for the spatial point process ‘Change in Type' (to model the recovery of individuals, and the loss of immunity). The complete closed system of equations describing the dynamics of *Q_S_*, *Q_I_* and *Q_R_* are presented in the electronic supplementary material, Supplementary Note S2. According to the definition of the modelling framework introduced in [49], the expected densities of individuals are given by an infinite perturbation series in the approximation of a long-ranged dispersal kernel, i.e. in the approximation of large *L*. We use only the leading and sub-leading contributions: the leading contribution is identical to a non-spatial analogue of this model, i.e. where the dispersal kernel is infinitely long-ranged (*L* = ∞); the sub-leading contribution becomes relevant when infection occurs over local spatial scales, i.e. the sub-leading contribution accounts for spatial and stochastic effects in a population of discrete individuals interacting over finite spatial scales.

### 2.2. Basic reproduction number and the invasion threshold

The basic reproduction number *R*_0_ is a powerful but also intuitively clear concept [33–36,38,39]. It defines the invasion threshold for a pathogen and hence for the invasion of disease: when *R*_0_ > 1 an initial introduction of a pathogen grows, and the pathogen invades the population causing an epidemic; when *R*_0_ < 1 an initial introduction of a pathogen decays. Also, in non-spatial models, *R*_0_ determines the so-called ‘herd immunity threshold' [40,41]: the pathogen cannot invade the population of *N* individuals where *N*(1 − 1/*R*_0_) individuals are immune to the infection. Herd immunity may be achieved by the accumulation of natural immunity or by vaccination. However, it has been shown that the estimates of *R*_0_ based on non-spatial models may break down when stochasticity and discreteness of individuals are taken into account [53].

*R*_0_ is defined as ‘the expected number of secondary cases produced by a typical infected individual during its entire period of infectiousness in a completely susceptible population' [33]. Thus, *R*_0_ can be calculated as ‘the product of the infection rate and the mean duration of the infection' [35]. Assuming the invasion of a pathogen occurs at time *t* = 0, the infection rate can be expressed as $Q_{I}^{-1}(0)\times [(dQ_{I}/dt){|}_{t=0}+\mu Q_{I}(0)]$, where the term in square brackets represents the rate of emergence of new infected individuals, which can be computed as the net rate of change of infected individuals (infections minus recoveries) plus the rate of recoveries; the pre-factor $Q_{I}^{-1}(0)$ guarantees that the infection rate is calculated for a unit initial introduction (in non-spatial systems an initial introduction corresponds to a single infected individual per whole finite population). Multiplying the infection rate by the infectious period 1/*μ* yields the basic reproduction number *R*_0_:

2.1 $$R_{0}=1+\frac{1}{\mu Q_{I}(0)}\frac{dQ_{I}(t)}{dt}{|}_{t=0}.$$

Expressions for *Q_I_*(0) and (d*Q_I_/dt*)|*_t_*_=0_ are non-trivial in spatially explicit models. In contrast with non-spatial models, a single infected individual within a spatially explicit model interacts not with the whole infinite-sized population, but only with individuals that can be contacted directly via a finite-ranged dispersal kernel, *b*(*x*). Even if mathematically the dispersal kernel *b*(*x*) is positive everywhere in infinite space, the infection transmissions over sufficiently large distances have an extremely low probability and therefore can be omitted from consideration. Thus, practically, the invasion is a localized phenomenon. Therefore, it is reasonable to consider a local neighbourhood area around an introduced infectious individual, such that all individuals within this neighbourhood area have a sufficiently high probability of having direct contact with the initially introduced infected individual during a single period of infectiousness. For clarity and in order to provide a mathematically transparent and intuitively clear estimate for *R*_0_, we define a neighbourhood area around an introduced infectious individual as a disk that includes a newly infected individual from that initial infected individual with probability *P* = 0.95. The radius L_*_ of the neighbourhood area is determined by the equation

2.2 $$\int_{0}^{L_{*}}2\pi rb(r)\;dr=0.95.$$

Note, the length L_*_ is proportional to the characteristic length scale, *L*, of the kernel, *b*(*x*), i.e. in the limit of large *L* the length L_*_ is also large. Next, considering the whole space as such that is covered by non-overlapping neighbourhood areas, the introduction of a single infected individual to each neighbourhood results in the initial density $Q_{I}(0)=1/(\pi \;L_{*}^{2})$. We assume that the total size of the population as well as the density, *n*, of the population are conserved; therefore, an infection can be introduced to each neighbourhood area by converting a single susceptible individual in each neighbourhood area into an infectious individual. Consequently, the initial values of other densities are as follows: *Q_S_*(0) = *n* − *Q_I_*(0), *Q_R_*(0) = 0, i.e. the total density equals *n*. The initial rate of change (d*Q_I_*/d*t*)|*_t_*_=0_ consists of leading and sub-leading contributions and is expressed straightforwardly from dynamical equations for these contributions, see electronic supplementary material, Supplementary Note S2. Substituting the formal expressions into the definition of *R*_0_ one obtains:

2.3 $$R_{0}=\frac{\beta N}{\mu }\left(1-\frac{1}{n\pi \;L_{*}^{2}}+\frac{\pi \;L_{*}^{2}}{n\mu }\int_{0}^{\infty }2\pi k\tilde{b}(k){\tilde{g}}_{SI}(k,0)\;dk\right),$$

where $\tilde{b}(k)$ and ${\tilde{g}}_{SI}(k,0)$ represent a Fourier transform of the dispersal kernel, *b*(*x*), and the second-order spatial cumulant *g_SI_*(*x*, *t*) between susceptible and infected individuals in the initial configuration in the system at time *t* = 0, respectively. We estimate ${\tilde{g}}_{SI}(k,0)$ approximately by constructing an auxiliary dynamical system where an equilibrium state has the same spatial structure as the initial condition in the main system, i.e. there is a single infected individual per neighbourhood area, on average. Details of this estimation and resulting analytical expression for ${\tilde{g}}_{SI}(k,0)$ are presented in electronic supplementary material, Supplementary Note S3. By exploring the dependence of expression (2.3) on the range *L* of dispersal kernel, we find that, in the limit of infinitely long range *L* → ∞, the only non-vanishing contribution is equal to the basic reproduction number in non-spatial models, *R*_0_ = *βN*/*μ*. Notice that equation (2.3) does not depend on the rate *γ* of the immunity loss; therefore, equation (2.3) is applicable for both SIR and SIRS models.

### 2.3. Endemic equilibrium and endemic threshold

For diseases with short-lived immunity, i.e. when *γ* > 0, an important characteristic of disease incidence is provided by the density of infected individuals at the endemic equilibrium, which we denote as $Q_{I}^{\mathrm{endemic}}$. The disease is endemic when $Q_{I}^{\mathrm{endemic}}>0$, and we refer to the condition $Q_{I}^{\mathrm{endemic}}=0$ as ‘the endemic threshold'. Within the analytical framework used in the current analysis, the analytical expression for $Q_{I}^{\mathrm{endemic}}$ is obtained as a stable fixed point of the corresponding dynamical equations for the densities of individuals. The resulting expression is shown below:

2.4 $$\begin{array}{rl} Q_{I}^{\mathrm{endemic}} & =\frac{\gamma /\mu }{\frac{\gamma }{\mu }+1}[n\left(\frac{R_{0}^{\left(0\right)}-1}{R_{0}^{\left(0\right)}}\right)-\int_{0}^{\infty }dk\frac{2\pi k{\tilde{b}}^{2}(k)}{{\left(R_{0}^{\left(0\right)}-\tilde{b}(k)\right)}^{2}}\;\\ & \times \begin{array}{c} \left(R_{0}^{\left(0\right)}-1)\gamma /\mu (\gamma /\mu +R_{0}^{\left(0\right)}+1)+R_{0}^{\left(0\right)}(R_{0}^{\left(0\right)}-\tilde{b}(k)\right)\\ \;+\;(1-\tilde{b}(k))(R_{0}^{\left(0\right)}\gamma /\mu +R_{0}^{\left(0\right)}-1)\;\\ \frac{}{\gamma /\mu (\gamma /\mu +R_{0}^{\left(0\right)})+(1+\gamma /\mu )(1-\tilde{b}(k))} \end{array}], \end{array}$$

where $R_{0}^{\left(0\right)}=\beta N/\mu$ is the basic reproduction number in non-spatial models.

### 2.4. Control measures

It is natural to classify many control interventions into spatial and non-spatial interventions based on their influence on spatially explicit pathogen transmission. Spatial control interventions reduce the distance over which individuals mix (e.g. the characteristic length scale *L* of the dispersal kernel *b*(*x*)), i.e. they reduce the number of contacts. However, we assume that this does not change the transmission rate at small distances, i.e. *βb*(0) = constant; figure 1*c*. Spatial interventions include many forms of restricted movement of individuals including quarantine. Within the context of the current pandemic of SARS-CoV-2, spatial interventions include lockdown [7,9,15] and social distancing acting on a large spatial scale, i.e. restriction of long-distance travelling [54,55]. Within agricultural systems, involving the spread of crop disease, spatial interventions include restriction of movement of inoculum, for example, on farm machinery [56], as well as quarantine in the movement of contaminated produce [57]. The ‘strength' of the spatial control is denoted by the parameter *s_L_*, where 0 ≤ *s_L_* ≤ 1. Thus, we model spatial control measures as such that transform model parameters in the following way:

2.5 $$\begin{array}{rl} & L\to (1-s_{L})L,\\ \mathrm{and}\;\;\;\; & \beta \to {\left(1-s_{L}\right)}^{2}\beta , \end{array}\}$$

where the scaling of *β* follows from the requirement *βb*(0) = constant and the normalization of the kernel *b*(*x*).

Non-spatial control measures reduce the infection rate, *β*; they do not change the characteristic length scale, *L*, of the dispersal kernel *b*(*x*); figure 1*c*. Within the context of an epidemic of SARS-CoV-2, and similar highly transmissible human pathogens, non-spatial control measures include using facemasks [4,5] and other personal protective equipment (PPE), good sanitation, hand washing, surface disinfecting [52,58], distancing between individuals by 1–2–3 m in public referred to as social distancing [31]. Contact tracing and subsequent isolation of infected people [2,3] can be considered as non-spatial control measures since this intervention does not reduce the distance over which undetected infectious individuals mix in the population. Within agricultural systems, non-spatial control measures include the use of pesticides and roguing (removal of symptomatically infected hosts). The ‘strength' of the non-spatial control is denoted by the parameter *s_β_*, 0 ≤ *s_β_* ≤ 1. Non-spatial control transforms the parameter *β* in the following way:

2.6 $$\beta \to (1-s_{\beta })\beta .$$

The impact of control measures on disease incidence is calculated by their impact on *R*_0_ and $Q_{I}^{\mathrm{endemic}}$ according to definitions (2.5) and (2.6), i.e. *R*_0_ → *R*_0_(*s_L_*,*s_β_*) and $Q_{I}^{\mathrm{endemic}}\to Q_{I}^{\mathrm{endemic}}(s_{L},s_{\beta })$. We incorporate vaccination into the model by removing a fraction of the population from the susceptible compartment. For example, when 70% of the population is vaccinated, 0.7*n* individuals per unit area are no longer susceptible and play no further role in the epidemic, so the initial spatial density of susceptible individuals is equal to 0.3*n*. We explore how the impact of control measures depends on the range of the dispersal kernel and the shape of the dispersal kernel (e.g. Gaussian or Tophat, or intermediate case; see electronic supplementary material, Supplementary Note S4 for further details).

### 2.5. Socio-economic cost of control measures

The socio-economic cost of applying control measures during a fixed time interval is assumed to be a function of the control strengths *s_β_* and *s_L_*. To demonstrate that an optimal control strategy depends on the relative cost of control measures, we consider three hypothetical scenarios with different costs for spatial and non-spatial control measures. Scenario (*a*): spatial control is moderately cheaper than non-spatial control. Scenario (*b*): spatial control is moderately more expensive than non-spatial control. Scenario (*c*): spatial control is much more expensive than non-spatial control. For illustration purposes, we choose the following cost functions:

2.7 $$\begin{array}{c} \mathrm{scenario}\;(a):\;\mathrm{cost}\;\mathrm{of}\;s_{L}=s_{L}^{2};\;\;\mathrm{cost}\;\mathrm{of}\;s_{\beta }=s_{\beta },\;\\ \mathrm{scenario}\;(b):\;\mathrm{cost}\;\mathrm{of}\;s_{L}=2s_{L}^{2};\;\mathrm{cost}\;\mathrm{of}\;s_{\beta }=s_{\beta }^{2},\;\\ \mathrm{scenario}\;(c):\;\mathrm{cost}\;\mathrm{of}\;s_{L}=2s_{L};\;\mathrm{cost}\;\mathrm{of}\;s_{\beta }=s_{\beta }^{2}.\; \end{array}\}$$

In each scenario (*a*)–(*c*), we considered all possible combinations of control measures with a constant total cost (equal to the sum of the cost of *s_L_* and the cost of *s_β_*) and identified a combination under which an invasion threshold is achieved with the minimal total cost. Details of the mathematical formulation of the problem and its solutions are shown in electronic supplementary material, Supplementary Note S5.

### 2.6. Parameters for numerical calculations

For numerical results, we used parameters of the COVID-19 epidemics caused by SARS-CoV-2 in the UK as the most relevant example: the population size *N* = 60 million corresponds to the approximate mainland GB population size; the basic reproduction number for SARS-CoV-2 $R_{0}^{\left(0\right)}=4$ [1], where the definition based on non-spatial models was used, $R_{0}^{\left(0\right)}=\beta N/\mu$; the average duration between onset of asymptomatic infectiousness and the end of infectiousness *μ*^−1^ = 4.4 days [5,59]; the infection rate per individual *β* = 0.15 × 10^−7^, inferred from the equation $R_{0}^{\left(0\right)}=\beta N/\mu$ using default values of *N*, $R_{0}^{\left(0\right)}$ and *μ*; the average duration between the end of infectiousness and the loss of immunity is assumed to be infinite for SARS-CoV-2, *γ*^−1^ = ∞, subject to the current absence of evidence for wide-spread loss of immunity to SARS-CoV-2. (We also considered *γ*^−1^ = 1 year which is consistent with data for other coronaviruses in human populations [60].) To study spatial effects, we assumed that individuals are distributed in space and interact with each other as follows: the average number of individuals per unit area *n* = 1; individuals are distributed randomly (Poisson process); individuals interact with each other via a Gaussian dispersal kernel, *b*(*x*), depending upon the distance, *x*, between interacting individuals, $b(x)=1/(2\pi L^{2})\exp(-x^{2}/(2L^{2}))$. The main results are shown using the length scale *L* = 3 (here *L* is measured in units where the population density is equal to 1); however, we also considered *L* = 1, 2, and *L* = ∞.

## 3. Results

First, to derive an accurate analytical description of epidemics in spatially explicit individual-based models, we used the unified framework for the analysis of individual-based models introduced in Cornell *et al.* [49]. We obtained the analytical expression for the basic reproduction number, *R*_0_, shown in equations (2.1)–(2.3); for diseases with short-lived immunity (*γ* > 0), we obtained the analytical expression for the density of infected individuals $Q_{I}^{\mathrm{endemic}}$ at the endemic equilibrium, equation (2.4).

Second, to quantify the relative impact of control interventions on epidemics, we classified control interventions as spatial and non-spatial based on their impact on dispersal kernel (figure 1*c*, equations (2.5) and (2.6)), and calculated *R*_0_ and $Q_{I}^{\mathrm{endemic}}$ for a range of possible combinations of spatial (with strength *s_L_*) and non-spatial (with strength *s_β_*) control measures. We used numerical values of parameters that correspond to ongoing COVID-19 epidemics in the UK, see results of calculations shown in figure 1*c,d*.

**Figure 2.**
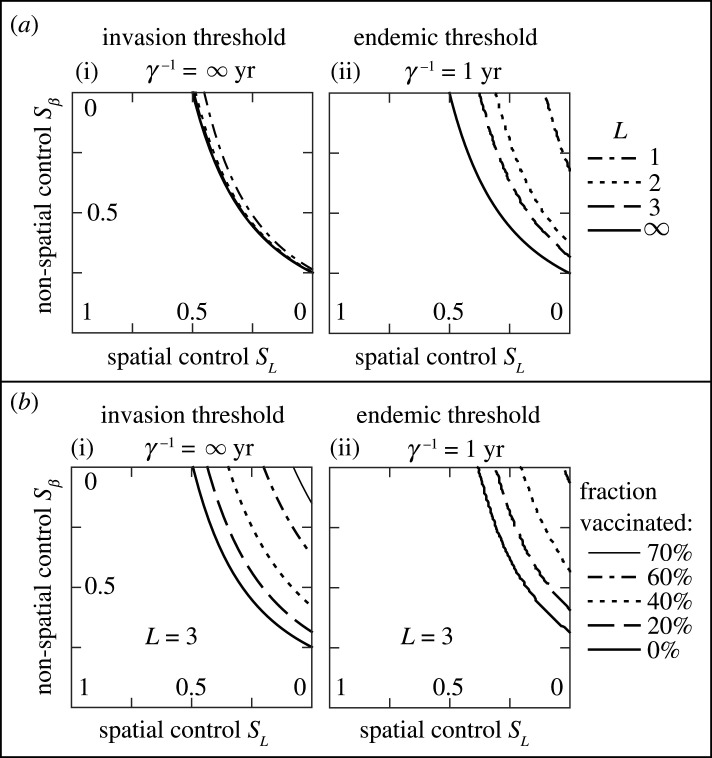
The trade-off between spatial and non-spatial control measures to achieve invasion and endemic thresholds. (*a*) The effect of the length scale, *L*, of the dispersal kernel *b*(*x*). Invasion thresholds at *L* > 1 appear visually indistinguishable. (*b*) The effect of vaccination of the specified fraction of the initial fully susceptible population. The vaccination determines the fraction of the population that is removed from the susceptible compartment and plays no further role in the epidemic.

To identify the optimal strategy for controlling a disease, we first defined a disease as being controlled when the invasion threshold *R*_0_(*s_L_*,*s_β_*) = 1 is achieved; an alternative definition for the case of endemic diseases is when the endemic threshold $Q_{I}^{\mathrm{endemic}}(s_{L},s_{\beta })=0$ is achieved. Next, we explored properties of these thresholds. We find that the thresholds for *R*_0_(*s_L_*,*s_β_*) = 1 and $Q_{I}^{\mathrm{endemic}}(s_{L},s_{\beta })=0$ differ in spatially explicit models (figure 1*e*). Thus, in spatially explicit models the pathogen (and hence disease) can invade a population, but then can fail to become endemic even when re-infection is possible, contrary to predictions for non-spatial models. This difference between thresholds occurs due to the lower density of susceptible individuals when the disease is endemic, making it less probable to find a susceptible individual near an infected individual. However, when the range of the dispersal kernel, *L*, increases, it is expected that the difference between invasion and endemic thresholds decreases and eventually vanishes. This was confirmed by our model: figure 2*a* where both thresholds coincide when *L* = ∞. Earlier studies [44,61] showed that in spatially explicit models where the host density is uniform, the invasion threshold is unchanged whether the interaction is short- or long-ranged. In line with those findings, we find only a weak dependence of the invasion threshold on *L* (figure 2*a*(i)). Such a dependence is weak because changing only *L* increases the local transmission rate due to the normalization of the dispersal kernel, thus the reduction in the number of contacts is compensated by the increase in the intensity of transmission. In contrast with the invasion threshold, the dependence of the endemic equilibrium on *L* is much stronger (figure 2*a*(ii)). This is again explained by the lower density of susceptible individuals at the endemic equilibrium. In principle, a disease can also be controlled by increasing the fraction of the population that has immunity to a pathogen, e.g. this can be achieved by vaccination. We have considered the trade-off of two types of control measures and the level of vaccination required to achieve invasion and endemic thresholds (figure 2*b*). Vaccination has a strong effect on both thresholds. Unsurprisingly, if a larger fraction of the population is vaccinated, then weaker control measures are required to control the disease. However, the inverse is practically useful when available vaccines or resources are limited: if stronger control measures are in place, then a lower fraction of the population needs to be vaccinated to control the disease. The shape of the dispersal kernel (e.g. Gaussian or Tophat, or intermediate case) has a weak effect on the invasion threshold, but a stronger effect on the endemic threshold, see electronic supplementary material, Supplementary Note S4 for further details.

**Figure 3.**
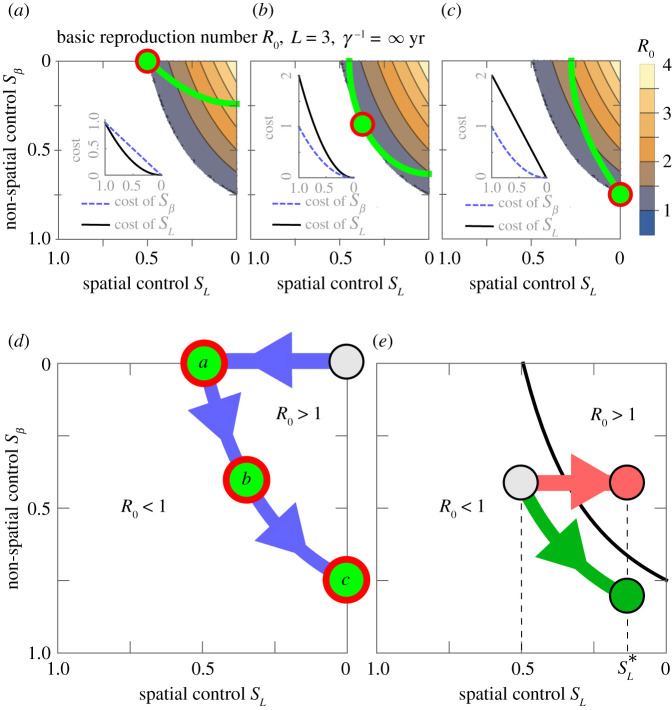
Optimal control strategies. (*a*–*c*) Optimal control in scenarios with different socio-economic cost of control measures defined in equation (2.7) and shown here as inset plots. The green curves are the curves of minimum total cost that intersect the *R*_0_ = 1 contour, the point of intersection (located in the centre of the red circle) provides the optimal combination of control interventions, i.e. optimal values for *s_L_* and *s_β_*. (*d*) Assuming socio-economic cost changes in time from scenario a to b to c, the optimal control strategy is expected to change in time as shown by blue arrows; (*e*) when reducing spatial control (such as exiting a lockdown strategy) the optimal strategy (green arrow) requires simultaneous increase of non-spatial control measures, while a non-optimal control strategy (red) keeps the strength of non-spatial control constant.

Finally, the identification of the optimal combination of spatial and non-spatial control interventions depends on the accurate estimation of the relative socio-economic cost of control interventions. We demonstrated this by using three hypothetical scenarios with different relative costs of control interventions (see equations (2.7)). The static optimal control strategy for each scenario is shown in figure 3*a–c*. It is highly likely that the optimal control strategy would change during the course of an epidemic, due to inevitable changes in the cost of control measures. To illustrate this, we assumed that the socio-economic cost of spatial control measures increases in time (e.g. due to the strong economic impact of lockdown on the economy). Correspondingly, we assume that the socio-economic cost of non-spatial control would slightly reduce in time (e.g. due to the development of new production facilities, or the increase in compliance with non-spatial control regulations). Under these circumstances, the time-dependent optimal combination of control interventions shifts towards using only non-spatial control measures (figure 3*d*), details are discussed in electronic supplementary material, Supplementary Note S5. If, eventually, the cost of spatial control measures, such as lockdown, become unacceptably high, lockdown may be relaxed or lifted entirely (i.e. decreasing the strength, *s_L_*, of spatial control measure). Our results show that the reduction of spatial control without a simultaneous increase of non-spatial control inevitably increases disease incidence by increasing the reproduction number; see the ‘red' strategy in figure 3*e*. Alternatively, it is possible to keep the reproduction number constant or even reduce it if non-spatial control interventions increase in strength while spatial control measures are being lifted; see the ‘green' strategy in figure 3*e*.

## 4. Discussion

We applied the unified framework of Cornell *et al.* [49] for the analysis of individual-based models to assess the optimal combination of spatial and non-spatial control interventions taking into account the socio-economic cost of control and the epidemiological impact on epidemics. In the current paper, we considered a simple example of a spatially distributed population of hosts. However, the methods of our paper could be used to model epidemics in populations in which individuals move or aggregate in clusters. The approach can also be applied to a wide class of diseases in populations of humans, animals and plants. For example, the methods we describe here can be readily applied to allow consideration of a large number of different types of individuals and interactions, therefore permitting analysis of, for example, stochastic multitype epidemics [37] of multiple infective strains [62] in heterogeneous spatially explicit populations with age structure [63] and different levels of mixing [63,64]. The work also contributes towards a new generation of analytical epidemic models that combine the following three characteristics that are necessary for realistic and reliable predictions: (i) stochasticity, (ii) spatially explicit dynamics and (iii) reliable mathematically controlled approximations.

The strong advantage of analytical models is that they provide insight and a general understanding of what combination of control measures would constitute an optimal control strategy taking account of the relative socio-economic cost of the measure. In addition, analytical models allow a rapid exploration of different intervention scenarios. This has a practical value: for example, the trade-off derived here between the two kinds of control measure and vaccination can suggest an optimal strategy for resource allocation. In particular, we showed that the application of stronger control measures reduces the level of vaccination required to achieve the herd immunity threshold [41,65]. Therefore, when the required number of doses of vaccine is not available or unacceptably costly, the application of stronger control measures can help to achieve the herd immunity threshold. This is valuable in the case of the current COVID-19 pandemic with the availability of vaccines [66]. The results of the methods explored here are also important in light of preparation for future epidemics especially in identifying ways to minimize the cost of future optimal control strategies [67–69].

## Data accessibility

The code for the model is released under the GNU Public License v. 2 and are available from Figshare at https://doi.org/10.6084/m9.figshare.13270265. To run the code, one needs to download the code for the underlying framework from https://www.nature.com/articles/s41467-019-12172-y.

## References

[1] Flaxman S et al. 2020 Estimating the effects of non-pharmaceutical interventions on COVID-19 in Europe. Nature **584**, 257-261. (10.1038/s41586-020-2405-7) 32512579

[2] Ferretti L, Wymant C, Kendall M, Zhao L, Nurtay A, Abeler-Dörner L, Parker M, Bonsall D, Fraser C. 2020 Quantifying SARS-CoV-2 transmission suggests epidemic control with digital contact tracing. Science **368**, eabb6936. (10.1126/science.abb6936) 32234805 PMC7164555

[3] Keeling MJ, Hollingsworth TD, Read JM. 2020 Efficacy of contact tracing for the containment of the 2019 novel coronavirus (COVID-19). J. Epidemiol. Community Health **74**, 861-866. (10.1136/jech-2020-214051) 32576605 PMC7307459

[4] Worby CJ, Chang HH. 2020 Face mask use in the general population and optimal resource allocation during the COVID-19 pandemic. Nat. Commun. **11**, 1-9. (10.1038/s41467-020-17922-x) 32792562 PMC7426871

[5] Stutt ROJH, Retkute R, Bradley M, Gilligan CA, Colvin J. 2020 A modelling framework to assess the likely effectiveness of facemasks in combination with ‘lock-down’ in managing the covid-19 pandemic. Proc. R. Soc. A **476**, 20200376. (10.1098/rspa.2020.0376) 32821237 PMC7428039

[6] Peak CM, Kahn R, Grad YH, Childs LM, Li R, Lipsitch M, Buckee CO. 2020 Individual quarantine versus active monitoring of contacts for the mitigation of COVID-19: a modelling study. Lancet Infect. Dis. **20**, 1025-1033. (10.1016/s1473-3099(20)30361-3) 32445710 PMC7239635

[7] Lau H, Khosrawipour V, Kocbach P, Mikolajczyk A, Schubert J, Bania J, Khosrawipour T. 2020 The positive impact of lockdown in Wuhan on containing the COVID-19 outbreak in China. J. Travel Med. **27**, 1-7. (10.1093/jtm/taaa037) PMC7184469 32181488

[8] Ferguson NM, Cummings DAT, Cauchemez S, Fraser C, Riley S, Meeyai A, Iamsirithaworn S, Burke DS. 2005 Strategies for containing an emerging influenza pandemic in Southeast Asia. Nature **437**, 209-214. (10.1038/nature04017) 16079797

[9] Ferguson NM, Cummings DAT, Fraser C, Cajka JC, Cooley PC, Burke DS. 2006 Strategies for mitigating an influenza pandemic. Nature **442**, 448-452. (10.1038/nature04795) 16642006 PMC7095311

[10] Yang Y, Sugimoto JD, Halloran ME, Basta NE, Chao DL, Matrajt L, Potter G, Kenah E, Longini Jr IM. 2009 The transmissibility and control of pandemic influenza A (H1N1). Science **326**, 729-733. (10.1161/CIRCULATIONAHA.110.956839) 19745114 PMC2880578

[11] Peak CM, Childs LM, Grad YH, Buckee CO. 2017 Comparing nonpharmaceutical interventions for containing emerging epidemics. Proc. Natl Acad. Sci. USA **114**, 4023-4028. (10.1073/pnas.1616438114) 28351976 PMC5393248

[12] Hill EM, Petrou S, De Lusignan S, Yonova I, Keeling MJ. 2019 Seasonal influenza: modelling approaches to capture immunity propagation. PLoS Comput. Biol. **15**, 1-26. (10.1371/journal.pcbi.1007096) PMC6837557 31658250

[13] Fong MW, Gao H, Wong JY, Xiao J, Shiu EYC, Ryu S, Cowling BJ. 2020 Nonpharmaceutical measures for pandemic influenza in nonhealthcare settings-international travel-related measures. Emerg. Infect. Dis. **26**, 976-984. (10.3201/eid2605.190995) 32027587 PMC7181936

[14] Lam HY et al. 2020 The epidemiology of COVID-19 cases and the successful containment strategy in Hong Kong—January to May 2020. Int. J. Infect. Dis. **98**, 51-58. (10.1016/j.ijid.2020.06.057) 32579906 PMC7306206

[15] Ferguson N et al. 2020 Report 9: impact of non-pharmaceutical interventions (NPIs) to reduce COVID-19 mortality and healthcare demand. See https://www.imperial.ac.uk/media/imperial-college/medicine/sph/ide/gida-fellowships/Imperial-College-COVID19-NPI-modelling-16-03-2020.pdf (10.25561/77482).

[16] Kantner M, Koprucki T. 2020 Beyond just ‘flattening the curve’: optimal control of epidemics with purely non-pharmaceutical interventions. J. Math. Ind. **10**, 1-23. (10.1186/s13362-020-00091-3) PMC7432561 32834921

[17] Scudellari M. 2020 The pandemic's future. Nature **584**, 22-25. 32760050 10.1038/d41586-020-02278-5

[18] Thompson RN et al. 2020 Key questions for modelling COVID-19 exit strategies. Proc. R. Soc. B **287**, 20201405. (10.1098/rspb.2020.1405) PMC7575516 32781946

[19] Davies NG et al. 2020 Effects of non-pharmaceutical interventions on COVID-19 cases, deaths, and demand for hospital services in the UK: a modelling study. Lancet Public Heal. **5**, e375-e385. (10.1016/S2468-2667(20)30133-X) PMC7266572 32502389

[20] López L, Rodó X. 2020 The end of social confinement and COVID-19 re-emergence risk. Nat. Hum. Behav. **4**, 746-755. (10.1038/s41562-020-0908-8) 32572175

[21] Rawson T, Brewer T, Veltcheva D, Huntingford C, Bonsall MB. 2020 How and when to end the COVID-19 lockdown: an optimization approach. Front. Public Heal. **8**, 262. (10.3389/fpubh.2020.00262) PMC7298102 32587844

[22] Ruktanonchai NW et al. 2020 Assessing the impact of coordinated COVID-19 exit strategies across Europe. Science **369**, 1465-1470. 32680881 10.1126/science.abc5096 PMC7402626

[23] Di Domenico L, Pullano G, Sabbatini CE, Boëlle PY, Colizza V. 2020 Impact of lockdown on COVID-19 epidemic in Île-de-France and possible exit strategies. BMC Med. **18**, 1-13. (10.1186/s12916-020-01698-4) 32727547 PMC7391016

[24] Aleta A et al. 2020 Modelling the impact of testing, contact tracing and household quarantine on second waves of COVID-19. Nat. Hum. Behav. **4**, 964-971. (10.1038/s41562-020-0931-9) 32759985 PMC7641501

[25] O'Sullivan D, Gahegan M, Exeter DJ, Adams B. 2020 Spatially explicit models for exploring COVID-19 lockdown strategies. Trans. GIS **24**, 967-1000. (10.1111/tgis.12660) PMC7283721 32837240

[26] Willem L, Verelst F, Bilcke J, Hens N, Beutels P. 2017 Lessons from a decade of individual-based models for infectious disease transmission: a systematic review (2006-2015). BMC Infect. Dis. **17**, 612. (10.1186/s12879-017-2699-8) 28893198 PMC5594572

[27] Hoertel N, Blachier M, Blanco C, Olfson M, Massetti M, Rico MS, Limosin F, Leleu H. 2020 A stochastic agent-based model of the SARS-CoV-2 epidemic in France. Nat. Med. **26**, 1417-1421. (10.1038/s41591-020-1001-6) 32665655

[28] Prem K et al. 2020 The effect of control strategies to reduce social mixing on outcomes of the COVID-19 epidemic in Wuhan, China: a modelling study. Lancet Public Heal. **5**, e261-e270. (10.1016/S2468-2667(20)30073-6) PMC7158905 32220655

[29] Keeling MJ. 1999 The effects of local spatial structure on epidemiological invasions. Proc. R. Soc. Lond. B **266**, 859-867. (10.1515/9781400841356.480) PMC1689913 10343409

[30] Hilton J, Keeling MJ. 2020 Estimation of country-level basic reproductive ratios for novel coronavirus (SARS-CoV-2/COVID-19) using synthetic contact matrices. PLoS Comput. Biol. **16**, 1-10. (10.1371/journal.pcbi.1008031) PMC7363110 32614817

[31] Block P, Hoffman M, Raabe IJ, Dowd JB, Rahal C, Kashyap R, Mills MC. 2020 Social network-based distancing strategies to flatten the COVID-19 curve in a post-lockdown world. Nat. Hum. Behav. **4**, 588-596. (10.1038/s41562-020-0898-6) 32499576

[32] Kermack WO, Mckendrick AG. 1927 A contribution to the mathematical theory of epidemics. Proc. R. Soc. A **115**, 700-721. (10.1098/rspa.1927.0118)

[33] Diekmann O, Heesterbeek JAP, Metz JAJ. 1990 On the definition and the computation of the basic reproduction ratio *R*_0_ in models for infectious diseases in heterogeneous populations. J. Math. Biol. **28**, 365-382. (10.1007/BF00178324) 2117040

[34] Hethcote HW. 2000 The mathematics of infectious diseases. SIAM Rev. **42**, 599-653.

[35] Van Den Driessche P, Watmough J. 2002 Reproduction numbers and sub-threshold endemic equilibria for compartmental models of disease transmission. Math. Biosci. **180**, 29-48. (10.1016/S0025-5564(02)00108-6) 12387915

[36] Heffernan JM, Smith RJ, Wahl LM. 2005 Perspectives on the basic reproductive ratio. J. R. Soc. Interface **2**, 281-293. (10.1098/rsif.2005.0042) 16849186 PMC1578275

[37] Britton T. 2010 Stochastic epidemic models: a survey. Math. Biosci. **225**, 24-35. (10.1016/j.mbs.2010.01.006) 20102724

[38] Cushing JM, Diekmann O. 2016 The many guises of *R*_0_ (a didactic note). J. Theor. Biol. **404**, 295-302. (10.1016/j.jtbi.2016.06.017) 27320680

[39] Delamater PL, Street EJ, Leslie TF, Yang YT, Jacobsen KH. 2019 Complexity of the basic reproduction number (*R*_0_). Emerg. Infect. Dis. **25**, 1-4. (10.3201/eid2501.171901) PMC6302597 30560777

[40] Fine P, Eames K, Heymann DL. 2011 ‘Herd immunity’: a rough guide. Clin. Infect. Dis. **52**, 911-916. (10.1093/cid/cir007) 21427399

[41] Randolph HE, Barreiro LB. 2020 Herd immunity: understanding COVID-19. Immunity **52**, 737-741. (10.1016/j.immuni.2020.04.012) 32433946 PMC7236739

[42] Bolker BM, Pacala SW, Levin SA. 2000 Moment methods for stochastic processes in continuous space and time. In The geometry of ecological interactions: simplifying spatial complexity (eds U Dieckmann, R Law, JAJ Metz), pp. 388–411. Cambridge studies in adaptive dynamics. Cambridge, UK: Cambridge University Press. (10.1017/CBO9780511525537.024)

[43] Bolker BM. 1999 Analytic models for the patchy spread of plant disease. Bull. Math. Biol. **61**, 849-874. (10.1006/bulm.1999.0115) 17886747

[44] Brown DH, Bolker BM. 2004 The effects of disease dispersal and host clustering on the epidemic threshold in plants. Bull. Math. Biol. **66**, 341-371. (10.1016/j.bulm.2003.08.006) 14871569

[45] Neuhauser C. 2002 Mathematical challenges in spatial ecology. Not. AMS **48**, 1304-1314. See https://www.ams.org/notices/200111/fea-neuhauser.pdf.

[46] Plank MJ, Law R. 2015 Spatial point processes and moment dynamics in the life sciences: a parsimonious derivation and some extensions. Bull. Math. Biol. **77**, 586-613. (10.1007/s11538-014-0018-8) 25216969

[47] Ovaskainen O, Cornell SJ. 2006 Space and stochasticity in population dynamics. Proc. Natl Acad. Sci. USA **103**, 12 781-12 786. (10.1073/pnas.0603994103) PMC1568924 16912114

[48] Ovaskainen O, Finkelshtein D, Kutoviy O, Cornell S, Bolker B, Kondratiev Y. 2014 A general mathematical framework for the analysis of spatiotemporal point processes. Theor. Ecol. **7**, 101-113. (10.1007/s12080-013-0202-8)

[49] Cornell SJ, Suprunenko YF, Finkelshtein D, Somervuo P, Ovaskainen O. 2019 A unified framework for analysis of individual-based models in ecology and beyond. Nat. Commun. **10**, 1-14. (10.1038/s41467-019-12172-y) 31624268 PMC6797757

[50] North AR, Godfray HCJ. 2017 The dynamics of disease in a metapopulation: the role of dispersal range. J. Theor. Biol. **418**, 57-65. (10.1016/j.jtbi.2017.01.037) 28130098 PMC5360276

[51] Atzrodt CL, Maknojia I, McCarthy RDP, Oldfield TM, Po J, Ta KTL, Stepp HE, Clements TP. 2020 A guide to COVID-19: a global pandemic caused by the novel coronavirus SARS-CoV-2. FEBS J. **287**, 3633-3650. (10.1111/febs.15375) 32446285 PMC7283703

[52] Meyerowitz EA, Richterman A, Gandhi RT, Sax PE. 2021 Transmission of SARS-CoV-2: a review of viral, host, and environmental factors. Ann. Intern. Med. **174**, 69-79. (10.7326/m20-5008) 32941052 PMC7505025

[53] Keeling MJ, Grenfell BT. 2000 Individual-based perspectives on *R*_0_. J. Theor. Biol. **203**, 51-61. (10.1006/jtbi.1999.1064) 10677276

[54] Kraemer MUG et al. 2020 The effect of human mobility and control measures on the COVID-19 epidemic in China. Science **368**, 493-497. (10.1126/science.abb4218) 32213647 PMC7146642

[55] Chinazzi M et al. 2020 The effect of travel restrictions on the spread of the 2019 novel coronavirus (COVID-19) outbreak. Science **368**, 395-400. (10.1126/science.aba9757) 32144116 PMC7164386

[56] Gilligan CA, Truscott JE, Stacey AJ. 2007 Impact of scale on the effectiveness of disease control strategies for epidemics with cryptic infection in a dynamical landscape: an example for a crop disease. J. R. Soc. Interface **4**, 925-934. (10.1098/rsif.2007.1019) 17609179 PMC1975768

[57] Parry M, Gibson GJ, Parnell S, Gottwald TR, Irey MS, Gast TC, Gilligan CA. 2014 Bayesian inference for an emerging arboreal epidemic in the presence of control. Proc. Natl Acad. Sci. USA **111**, 6258-6262. (10.1073/pnas.1310997111) 24711393 PMC4035939

[58] Jefferson T et al. 2011 Physical interventions to interrupt or reduce the spread of respiratory viruses. Cochrane Database Syst. Rev. **2011**, CD006207. (10.1002/14651858.CD006207.pub4) PMC6993921 21735402

[59] He X et al. 2020 Temporal dynamics in viral shedding and transmissibility of COVID-19. Nat. Med. **26**, 672-675. (10.1038/s41591-020-0869-5) 32296168

[60] Edridge AWD et al. 2020 Seasonal coronavirus protective immunity is short-lasting. Nat. Med. **26**, 1691-1693. (10.1038/s41591-020-1083-1) 32929268

[61] Holmes EE. 1997 Basic epidemiological concepts in a spatial context. In Spatial ecology: the role of space in population dynamics and interspecific interactions (eds D Tilman, P Kareiva), Princeton, NJ: Princeton University Press.

[62] Gog JR, Grenfell BT. 2002 Dynamics and selection of many-strain pathogens. Proc. Natl Acad. Sci. USA **99**, 17 209-17 214. (10.1073/pnas.252512799) PMC139294 12481034

[63] Britton T, Ball F, Trapman P. 2020 A mathematical model reveals the influence of population heterogeneity on herd immunity to SARS-CoV-2. Science **369**, 846-849. (10.1126/science.abc6810) 32576668 PMC7331793

[64] Ball F, Mollison D, Scalia-Tomba G. 1997 Epidemics with two levels of mixing. Ann. Appl. Probab. **7**, 46-89. (10.1214/aoap/1034625252)

[65] Fontanet A, Cauchemez S. 2020 COVID-19 herd immunity: where are we? Nat. Rev. Immunol. **20**, 583-584. (10.1038/s41577-020-00451-5) 32908300 PMC7480627

[66] Krammer F. 2020 SARS-CoV-2 vaccines in development. Nature **586**, 516-527. (10.1038/s41586-020-2798-3) 32967006

[67] Bedford J, Farrar J, Ihekweazu C, Kang G, Koopmans M, Nkengasong J. 2019 A new twenty-first century science for effective epidemic response. Nature **575**, 130-136. (10.1038/s41586-019-1717-y) 31695207 PMC7095334

[68] Saad-Roy CM et al. 2020 Immune life history, vaccination, and the dynamics of SARS-CoV-2 over the next 5 years. Science **21**, 1-9. (10.1126/science.abd7343) PMC7857410 32958581

[69] Metcalf CJE, Morris DH, Park SW. 2020 Mathematical models to guide pandemic response. Science **369**, 368-370. (10.1126/science.abd166824) 32703861

