# Correction: ‘Analytical approximation for invasion and endemic thresholds, and the optimal control of epidemics in spatially explicit individual-based models’ (2021), by Suprunenko *et al.*

**DOI:** 10.1098/rsif.2024.0866

**Published:** 2025-01-15

**Authors:** Yevhen F. Suprunenko, Stephen J. Cornell, Christopher A. Gilligan

**Affiliations:** ^1^ Department of Plant Sciences, University of Cambridge, Cambridge, UK; ^2^ Institute of Infection, Veterinary, and Ecological Sciences, University of Liverpool, Liverpool, UK

*J. R. Soc. Interface* **18**, 20200966. (Published online 31 March 2021) (https://doi.org/10.1098/rsif.2020.0966).

The authors note that equation (2.3) in the main text and equation (19) in electronic supplementary material appeared incorrectly. On the right-hand side of both equations inside the brackets in the last term there should be no μ in the denominator. The corrected equations appear below.


(2.3) $$R_{0}=\frac{\beta N}{\mu }\left(1-\frac{1}{n\pi \;L_{*}^{2}}+\frac{\pi \;L_{*}^{2}}{n}\int_{0}^{\infty }2\pi k\tilde{b}\left(k\right){\tilde{g}}_{SI}\left(k,0\right)dk\right),$$

(19) $$\begin{array}{rl} f\left(s_{L}\right)=\; & 1-\frac{\mu }{\beta N{\left(1-s_{L}\right)}^{2}}(1-\frac{1}{n\pi {\left(1-s_{L}\right)}^{2}L_{*}^{2}}+\\ & {+\frac{\pi {\left(1-s_{L}\right)}^{2}L_{*}^{2}}{n}\int_{0}^{\infty }2\pi k\tilde{b}\left(k;s_{L}\right){\tilde{g}}_{SI}\left(k,0;s_{L}\right)dk)}^{-1} \end{array}$$

Correct forms of the equations were used in all computations and the results and conclusions remain unchanged.

